# Investigation of interleukin-2-mediated changes in blood pressure, fetal growth restriction, and innate immune activation in normal pregnant rats and in a preclinical rat model of preeclampsia

**DOI:** 10.1186/s13293-020-00345-0

**Published:** 2021-01-06

**Authors:** Mark W. Cunningham, Lorena M. Amaral, Nathan E. Campbell, Denise C. Cornelius, Tarek Ibrahim, Venkata Ramana Vaka, Babbette LaMarca

**Affiliations:** 1 Department of Pharmacology & Toxicology, Center for Excellence in Renal and Cardiovascular Research, University of Mississippi Medical Center, Jackson, MS 39216 USA; 2 Department Of Emergency Medicine, University of Mississippi Medical Center, Jackson, MS USA; 3 Division of Maternal Fetal Medicine, Department Of Obstetrics and Gynecology, University of Mississippi Medical Center, Jackson, MS 39216 USA

## Abstract

Two important clinical features of preeclampsia (PE) are hypertension and fetal growth restriction. The reduced uterine perfusion pressure (RUPP) preclinical rat model of PE exhibits both of these features. Moreover, RUPP and PE women have elevated vasoconstrictor peptide endothelin-1 (ET-1) and inflammation. Interleukin-2 (IL-2) is a cytokine that regulates NK cell activity and is elevated in miscarriage, PE, and RUPP rats. The objective of this study was to examine a role for IL-2 in NK cell activation, fetal growth restriction, and hypertension during pregnancy by either infusion of IL-2 or blockade of IL-2 (basiliximab) in normal pregnant (NP) and RUPP rats. On gestational day 14, NP and RUPP rats received low (LD), middle (MD), or high dose (HD) IL-2 (0.05, 0.10, or 0.20 ng/ml) IP or basiliximab (0.07 mg per rat) by IV infusion. On day 19, blood pressure (MAP), pup weights, and blood were collected. Basiliximab had no effect on blood pressure, however, significantly lowered NK cells and may have worsened overall fetal survival in RUPP rats. However, IL-2 LD (102 ± 4 mmHg) and IL-2 HD (105 ± 6 mmHg) significantly lowered blood pressure, ET-1, and activated NK cells compared to control RUPPs (124 ± 3 mmHg, *p* < 0.05). Importantly, IL-2 in RUPP rats significantly reduced fetal weight and survival. These data indicate that although maternal benefits may have occurred with low dose IL-2 infusion, negative effects were seen in the fetus. Moreover, inhibition of IL-2 signaling did not have favorable outcome for the mother or fetus.

## Introduction

Preeclampsia (PE) is a multi-system disorder characterized by new onset hypertension during pregnancy [1–6]. PE is associated with increased cytokines, natural killer (NK) cells, T-helper 1 (TH1) cells, TH17 cells, and a decrease in T regulatory (T regs) and TH2 cells [2, 3, 6]. The reduced uterine perfusion pressure (RUPP) rat model of placental ischemia is a well-established model of PE that recapitulates many of the pathophysiological characteristics of PE. Previous studies by our group and others have demonstrated the importance of TH1 cells and NK cells in mediating pathophysiology in response to RUPP that is similar to that observed in women with PE [6–9].

NK cells are an important part of innate immunity and can be stimulated by a decrease in major histocompatibility complex class 1 (MHC I) self-proteins which activate killing signals toward tumor cells or virus-infected cells. Natural killer cells can also be influenced by memory CD4^+^ TH1 cells through their secretion of interleukin-2 (IL-2) which is elevated in preeclamptic women and associated with other pathological conditions of pregnancies [10–14]. Cytolytic NK cell population has been shown to be increased in patients with preeclampsia and miscarriage versus healthy control patients. Healthy pregnancy is also associated with higher uterine NK cells within their placentas than PE patients. We believe that pathophysiology and adverse outcomes seen in PE may be in part due to the increase in cytolytic NK cells and decrease in uterine NK cells. Importantly, uterine NK cells, which are noncytolytic in nature, are characterized by their release of the anti-inflammatory cytokines IL-5 and IL-13 [15, 16], and they communicate with T reg cells to create a feto-tolerant uterine environment [15–18].

IL-2 plays an important role in establishing T cell memory and potentiates NK cell cytolytic activity and proliferation. IL-2 is primarily known as a T cell growth factor that has both autocrine and paracrine actions and binds to CD25 on T cells [19–21]. T regs respond to secreted IL-2 from effector T cells and send signals to decrease the effector T cell proliferation and their secretion of IL-2. Therefore, T regs can inhibit endogenous IL-2 production and thus control IL-2-mediated differentiation of NK cells [15, 17, 22]. We have previously shown that IL-2 is increased in RUPP rats [18]. Moreover, we have demonstrated that stimulating endogenous T regs with superagonistic (SA) monoclonal antibody for CD28 (JJ316) in RUPP rats lowered circulating IL-2 and increased endogenous T regs [23]. In addition, this increase in T regs resulted in lowered endothelin (ET-1), a vasoconstrictor peptide, in the placenta and kidney of RUPP rats, which was associated with lowered blood pressure in the treated RUPPS. However, this technique led to death in clinical trials by stimulating a cytokine storm and is questionable as a future therapeutic. Therefore, targeting IL-2 doses in many different disease states has been the foci of many clinical studies.

Multiple case studies have examined the dose-dependent effect of IL-2 in patients with various carcinomas or in other animal models of disease [24–27]. These studies demonstrate the effect of increasing doses of IL-2 on T regs, effector T cells, and natural killer cells to improve disease outcome. Low dose IL-2 (0.3 × 106 to 3.0 × 106 IU) was shown to cause a 75% increase in T regs in the blood of patients with ischemic heart disease and thus considered that the safe and tolerable dose for diseases in which increased T regs would improve clinical outcome, such as chronic inflammatory states [24–27]. This increase in T regs has been associated with a reduction in the proinflammatory response and improvement in clinical feature of disease in patients with type 1 diabetes and in patients with autoimmune liver disease, lupus, and in ischemic heart disease [24–27].

High doses of IL-2, like that resulting from cytokine storm, cause capillary damage and extravasation of fluid, renal and liver damage, and hypotension [24–27]. However, higher doses of IL-2 have been used in combination with other drugs as a chemotherapeutic agent [24–27]. It has proven effective against many metastatic cancers such as metastatic melanoma and renal cell carcinoma [24–27]. High dose IL-2 treatment is advantageous in treating metastatic cancer due to heightened activity of natural killer cells toward the tumors. These dosing regimens are much more acceptable and common and have been approved for treatment for a much longer timeframe than those utilizing low dose IL-2 regimens.

Since IL-2 is elevated in RUPP rats, we hypothesized that the increase in IL-2 might be driving the hypertension and reduced fetal growth. Our first attempt to control IL-2 was influenced by the aforementioned clincial studies invoking the low dose IL-2 regimen to activate T regs and improve a chronic inflammatory response; we infused low dose IL-2 (3 regimens between 0.01 and 0.05 IU) into the RUPP rat and evaluated its effect on the observed clincial features of PE. Our next objective of this study was to determine if blockade of IL-2 with basiliximab (Simulect), a chimeric monoclonal antibody that functions as an immunosuppressive agent, could reduce hypertension in RUPP rats by lowering ET-1 and improve pup weight by inhibiting activated NK cells.

## Methods

Timed-pregnant Sprague Dawley (SD) rats purchased from Envigo (Indianapolis, IN) were used in this study. Rats were housed in a temperature-controlled room (75 °F) with a 12 h light and dark cycle each day and maintained on a normal diet with free access to food and water. All experiments were performed in accordance with the National Institutes of Health guidelines for use and care of animals, and all animal protocols were approved by the Institutional Animal Care and Use Committee (IACUC) at the University of Mississippi Medical Center.

### Effect of IL-2 on blood pressure during pregnancy

Our first objective of the study was to examine the effect of three different low dose regimens of IL-2 on NK cell activation and mean arterial pressure (MAP) on RUPP and control (NP) rats and compare outcomes to control NP and RUPP groups. Although all are within the low dose range, they are identified as low dose, middle dose, and high dose. Rats were divided into NP + LD IL-2 (*n* = 9), NP + MD IL-2 (*n* = 5), NP + HD IL-2 (*n* = 12), RUPP + LD IL-2 (*n* = 7), RUPP + MD IL-2 (*n* = 4), and RUPP + HD IL-2 (*n* = 4). Recombinant rat IL-2 (R and D Systems; 6502-RL, Minneapolis, MN) was delivered at 3 different doses; a low dose (LD = 0.01 IU) 0.05 ng/ml, middle dose (MD = 0.02) 0.10 ng/ml, and a high dose (HD = 0.05 IU) 0.20 ng/ml were infused intraperitoneal by a miniosmotic pump (Alzet; Model 2002) inserted on day 14 of pregnancy. On day 14 of pregnancy, the RUPP surgery was performed as previously described [1, 5–7, 28]. In order to test the effect of IL-2 blockade on hypertension and fetal weight, basiliximab (Simulect), a monoclonal antibody to the α-chain of the IL-2 receptor that blocks IL-2 signaling, was administered once via IV at a dose of 0.07 mg per rat into two groups of rats (NP and RUPP rats) on day 14 of gestation. This dose was chosen to mimic the administration of basiliximab in adult human patients. On day 18 of gestation, under isoflurane anesthesia, catheters were inserted into the carotid artery of all rats to measure the blood pressure. On day 19 of gestation, conscious blood pressures (MAP) were determined; blood, pup weights, placentas, and kidneys were collected, weighed, processed for flow cytometry, or stored in − 80 °C for further analysis [29, 30]. All placentas and pups were weighed, and the average weight for the placenta and pup was calculated by adding all of placenta and pup weights from each litter (pregnant rat) and then dividing by the number of placentas/pups per litter. The average of each litter was placed in each respectable animal group and used to determine the average weight for the placenta and pups per group. For flow cytometry, whole blood was collected at room temperature, and tissues were collected in tubes with PBS.

Whole blood was collected in serum separator tubes at room temperature and left to set for up to 2 h and centrifuged at 3200 rpm for 10 min at 4 °C. The resulting supernatant is stored as serum at − 20 °C. Plasma is collected in ice cold EDTA-containing tubes, centrifuged for 10 min at 3200 rpm at 4 °C and stored the plasma at − 20 °C.

### Determination of circulating and placental natural killer (NK) cells and circulating T regulatory cells by flow cytometry

Lymphocytes were isolated from blood and tissues via centrifugation on a cushion of Ficoll-Hypaque (Lymphoprep, Accurate Chemical & Scientific Corp., Westbury, NY). For flow cytometry, 1 × 10^6^ cells were incubated for 10 min at 4 °C with antibodies against rat natural killer cell markers ANK61 and ANK44 (AbCam, Cambridge, MA). ANK61 binds to an antigen that is expressed on all NK cells, while the antigen for ANK44 is only expressed on stimulated, cytotoxic NK cells [22]. After washing, cells were labeled with secondary Fluorescein isothiocyanate (FITC; AbCam) antibody for 10 min at 4 °C. For staining of regulatory T cells, 1 × 10^6^ cells were incubated for 10 min at 4 °C with anti-rat CD4-FITC and CD25-PE (BD Bioscience, San Jose, CA). Cells were then washed, permeabilized, and stained with anti-rat FOXp3-APC (RnD Systems, Minneapolis, MN). As a negative control for each individual rat, cells were treated exactly as described above except they were incubated with isotype control antibodies conjugated to FITC, PE, or APC as appropriate. Subsequently, cells were washed, fixed, and resuspended in 500 μL of Roswell Park Memorial Institute medium (RPMI) and run on the Miltenyi MACSQuant Analyzer 10 (San Diego, CA) after compensation, and analyzed using the FlowLogic software (Innovai, Sydney, Australia). Lymphocytes were gated in the forward and side scatter plots, and doublet exclusion was performed. Cells that stained as ANK61+ were designated as NK cells. Cells that stain as ANK44+ were designated as activated NK cells. Cells that stained CD4+/CD25+/FoxP3+ were designated as T regulatory cells. The percent of positive-stained cells above the negative control was determined for individual rats, and the mean values for each experimental group were calculated.

### Determination of endothelin-1 by real time PCR

Real-time PCR was used to determine levels of placental and renal PPET-1 (preproendothelin-1) as previously described [12]. Total RNA from the placenta or renal cortex was extracted using the RNeasy Protect Mini kit (Qiagen, Hilden, Germany). cDNA was synthesized from 1 μg of RNA with Bio-Rad iScript cDNA reverse transcriptase, and real-time PCR was performed using the Bio-Rad SYBR Green supermix (Bio-Rad, Hercules, CA). PPET-1 expression is calculated by ΔΔCT method using B actin as the house keeping gene and expressed as the fold difference from NP rats.

### Determination of sFlt-1

Plasma collected from all pregnant rats was measured for sFlt-1 levels using commercial ELISA kits from R&D Systems (Quantikine) according to the manufacturer’s instructions. The minimal detectable levels for sFlt-1 were 15.2 pg/mL, with inter- and intra-assay variability of 8.4% and 7.2%.

### Statistical analysis

All data are presented as mean ± SEM. All data were found to be normally distributed and analyzed by two-way ANOVA with Bonferroni post hoc analysis. Preproendothelin-1 expression was analyzed by one-way ANOVA with Bonferroni post hoc test. Flow cytometry data were found to be normally distributed and analyzed by one-way ANOVA with Bonferroni post hoc analysis or by Student’s *t* test when comparing NP + basiliximab vs RUPP + basiliximab treatment and T reg levels between RUPP and RUPP+IL-2 at any dose. All statistical analysis was performed with the Graphpad Prism 7 software (GraphPad Software, La Jolla, CA). *p* < 0.05 was considered statistically significant.

## Results

### The effect of IL-2 infusion on blood pressure and fetal weights in RUPP and NP rats

**Fig. 1:**
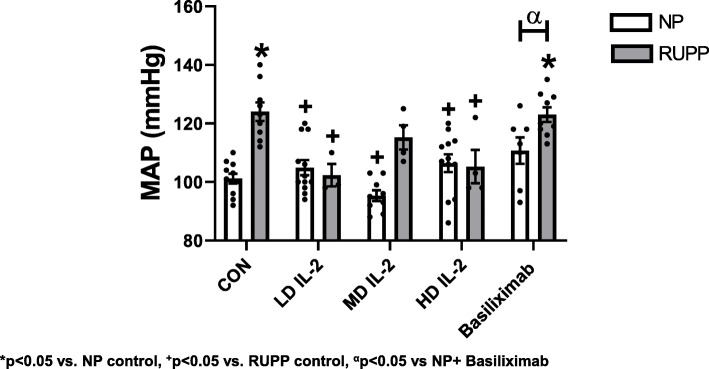
The mean arterial pressure (MAP) for each group. MAP was elevated in RUPP (*n* = 9) vs. normal pregnant rats (NP) (*n* = 11). Low (LD) (*n* = 3) and high (HD) (*n* = 4) dose IL-2 treatment in RUPP rats decreased MAP in comparison to RUPPs. The middle (MD) (*n* = 4) dose of IL-2 had a similar MAP as NP rats. Basiliximab treatment had no effect on NP and RUPP MAP. RUPP + basiliximab (*n* = 9) treatment increased MAP in comparisons to NP and NP + basiliximab (*n* = 7) treatment. Statistical differences were achieved using two-way ANOVA with Bonferroni post hoc analysis, *p < 0.05 vs NP control and ^+^*p* < 0.05 vs. RUPP control. Student *t test* was also used in comparing NP + basiliximab vs. RUPP + basiliximab, ^α^*p* < 0.05 vs NP + basiliximab

**Fig. 2:**
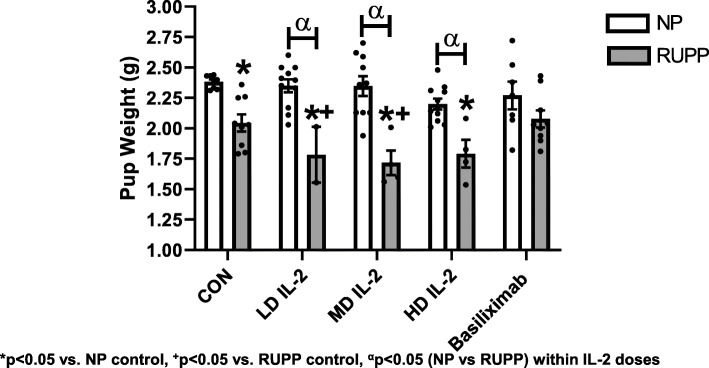
The pup weight for NP (*n* = 8), NP + LD (*n* = 11), NP + MD (*n* = 10), NP + HD (*n* = 11), NP + basiliximab (*n* = 7), RUPP (*n* = 9), RUPP + LD (*n* = 3), RUPP + MD (*n* = 4), RUPP + HD (*n* = 4), and RUPP + basiliximab (*n* = 9). All pup weights were reduced in RUPP rats compared to NP rats with or without IL-2 infusion at all 3 doses (LD, MD, HD). Statistical differences were achieved using two-way ANOVA with Bonferroni post hoc analysis, *p < 0.05 vs. NP control, ^+^*p* < 0.05 vs. RUPP control, ^α^*p* < 0.05 (NP vs RUPP) within IL-2 doses

MAP was significantly elevated in RUPP vs. NP rats (124 ± 3 vs. 101 ± 2 mmHg, *p* < 0.05) (Fig. 1), and significantly decreased in RUPP rats treated with the LD IL-2 (102 ± 4 mmHg, *p* < 0.05 vs. RUPP) and HD IL-2 (105 ± 6 mmHg, *p* < 0.05 vs. RUPP, Fig. 1). Blood pressure was lower in RUPP rats treated with the middle dose (MD IL-2) (115 ± 4 vs. 124 ± 3 mmHg, *p* > 0.99); however, this pressure was not significantly less than the pressure observed in RUPP rats. IL-2 infusion at the LD (105 ± 3 mmHg), MD (95 ± 2 mmHg), and HD (106 ± 3 mmHg) in NP rats did not change MAP compared to NP control (101 ± 2 mmHg) rats (*p* > 0.99 for all IL-2 doses in NP vs NP control) (Fig. 1). Pups (2.04 ± 0.07 vs. 2.38 ± 0.02 g) and placental weight (0.53 ± 0.04 vs. 0.66 ± 0.02 g) were decreased in RUPP vs NP rats (*p* < 0.05 vs. NP, Fig. 2). Importantly, the pup weight of RUPPs treated with IL-2 were lower than RUPP control pup weight (*p* < 0.05) (Fig. 2). All RUPP rats had an increase in percent reabsorptions and decrease in percent survival, despite the IL-2 dose administered (*p* < 0.05) (Table 1), with RUPP + MD (*p* = 0.43) and RUPP + HD IL-2 (*p* = 0.33) increasing fetal death to a much greater degree than RUPP alone; however, this did not quite reach statistical significance.

**Table 1:**
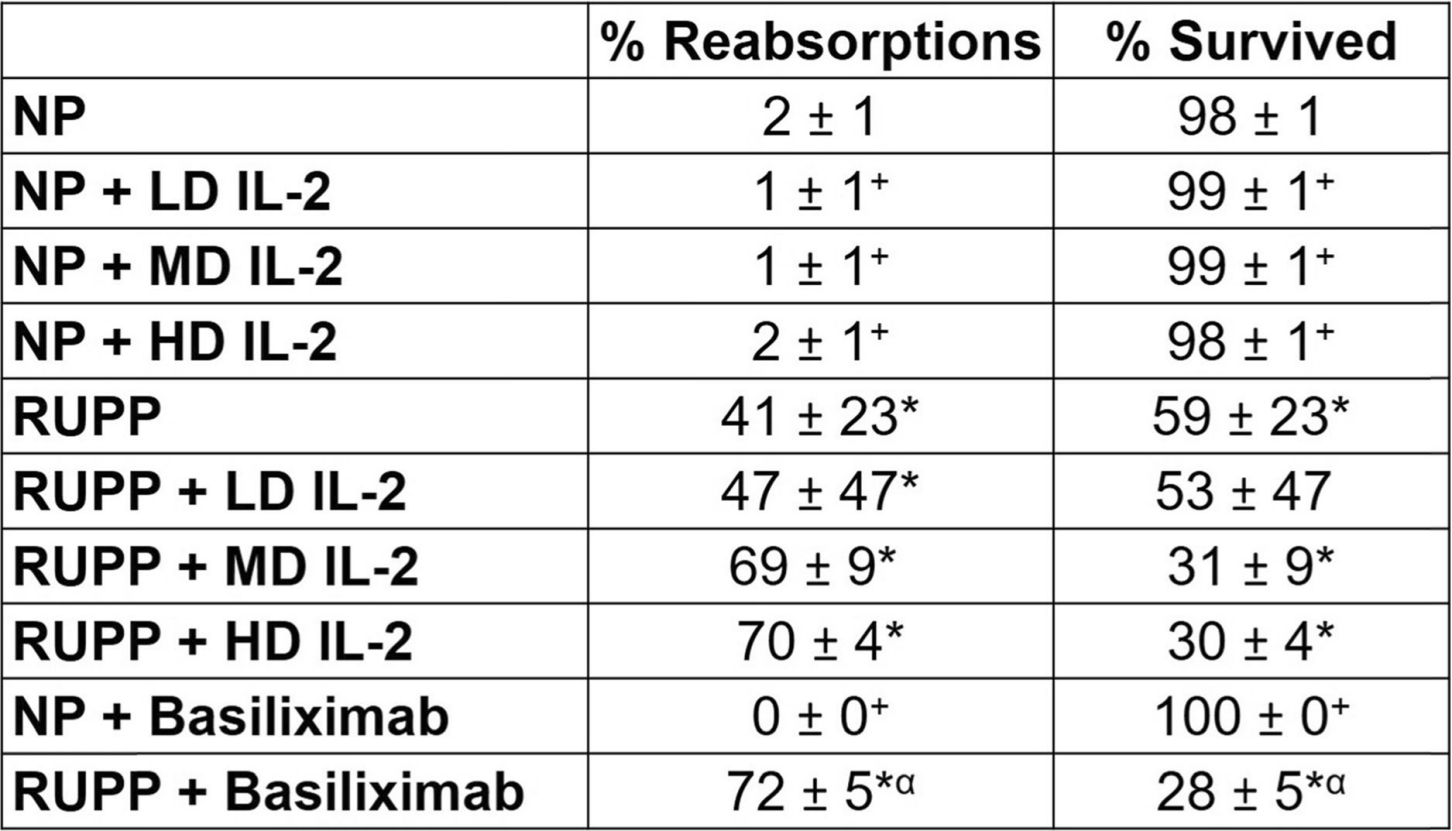
Percent fetal reabsorption and survivability

*p < 0.05 vs NP control; ^+^*p* < 0.05 vs RUPP control; ^α^*p* < 0.05 (NP + basiliximab vs RUPP + basiliximab)

### The effect of IL-2 infusion on T regs and natural killer cells in RUPP and NP rats

**Fig. 3:**
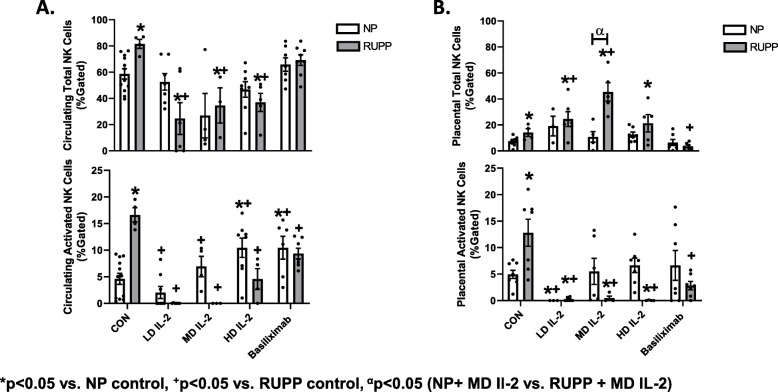
**a** Circulating total and activated NK cells in NP (*n* = 12), RUPP (*n* = 4), RUPP + LD IL-2 (*n* = 6), RUPP + MD IL-2 (*n* = 3), RUPP + HD IL-2 (*n* = 5), NP + basiliximab (*n* = 7), and RUPP + basiliximab (*n* = 8). Circulating total and activated NK cells were elevated in RUPP vs NP rats. All doses of IL-2 administered to RUPP rats decreased circulating NK cells in comparison to RUPP rats. Basiliximab increased circulating activated NK cells in NP rats, while it reduced circulating activated NK cells in RUPP rats. b Placental total and activated NK cells in NP (*n* = 10), RUPP (*n* = 4), RUPP + LD IL-2 (*n* = 6), RUPP + MD IL-2 (*n* = 5), RUPP + HD IL-2 (*n* = 5), NP + basiliximab (*n* = 7), and RUPP + basiliximab (*n* = 7). Placental NK cells were elevated in RUPP vs NP rats. In addition, total placental NK cells were elevated with all doses of IL-2 in RUPP rats; however, activated NK cells were significantly reduced with IL-2 in RUPPs. RUPP + basiliximab treatment reduced placental total NK cells compared to RUPP rats. Statistical differences were achieved using two-way ANOVA with Bonferroni post hoc analysis, *p < 0.05 vs. NP control, ^+^*p* < 0.05 vs. RUPP control, ^α^*p* < 0.05 (NP + MD Il-2 vs. RUPP + MD IL-2)

T regs were 4.0 ± 1.0 in NP vs 1.3 ± 0.55% gated in RUPP rats (*p* < 0.05; *t* test) (data not shown). T regs increased with each dose of IL-2 given to RUPP rats. LD IL-2 increased T regs to 4.0 ± 2.0% (*p* < 0.05); MD IL-2 increased T regs to 12.83 ± 7% gated in RUPP rats; (*p* < 0.05 vs RUPP; *t* test); HD IL-2 increased T regs to 12.96 ± 5% gated in RUPP rats; (*p* < 0.05 vs RUPP; *t* test) (data not shown). Compared to NP controls, total and activated circulating NK cells in RUPP are significantly elevated and were lowered with all three doses of IL-2 administered to RUPP rats (*p* < 0.05) (Fig. 3a). Total and cytolytic NK cells were elevated in placentas of RUPP vs. NP rats (*p* < 0.05). Interestingly, total placental NK cells are elevated with all doses of IL-2 in RUPP rats, yet IL-2 decreased placental activated (cytolytic) NK cells in comparison to both NP and RUPP rats (*p* < 0.05) (Fig. 3b). There were no changes in circulating or placental total and activated NK cells between NP and NP + IL-2-treated rats.

### The effect of IL-2 infusion on preproendothelin expression in the placenta and kidney during pregnancy

**Fig. 4:**
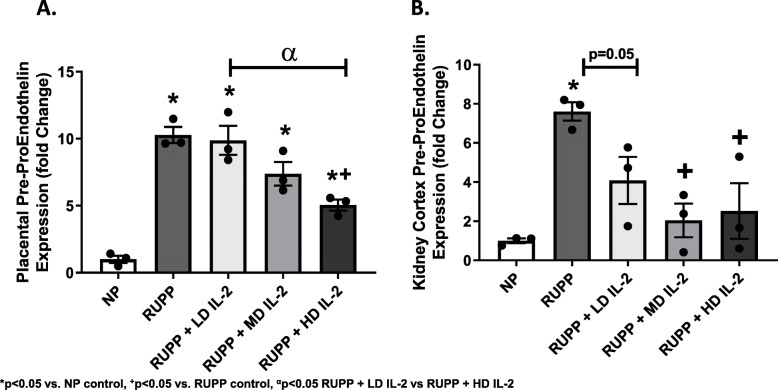
**a** Placental preproendothelin expression in NP (*n* = 3), RUPP (*n* = 3), RUPP + LD IL-2 (*n* = 3), RUPP + MD IL-2 (*n* = 3), and RUPP + HD IL-2 (*n* = 3). Placental preproendothelin (PPET) was elevated in RUPP compared to NP rats. There was a significant reduction in placental PPET between RUPP + HD IL-2 and RUPP rats. **b** Renal cortex preproendothelin expression in NP (*n* = 3), RUPP (*n* = 3), RUPP + LD IL-2 (*n* = 3), RUPP + MD IL-2 (*n* = 3), and RUPP + HD IL-2 (*n* = 3). Renal cortex PPET is elevated in RUPP vs. NP rats. All doses of IL-2 reduced PPET expression in comparison to RUPP rats. Statistical differences were achieved using one-way ANOVA with Bonferroni post hoc analysis,*p < 0.05 vs NP control, ^+^*p* < 0.05 vs. RUPP control, ^α^*p* < 0.05 RUPP + LD IL-2 vs RUPP + HD IL-2

Preproendothelin (PPET) is elevated 8–10-folds in the kidney and placenta of RUPP vs. NP rats (*p* < 0.05) (Fig. 4a). Importantly, IL-2 infusion significantly lowered PPET expression in the renal cortex for all doses and in placentas of RUPP rats at the LD IL-2 (*p* > 0.99), MD IL-2 (*p* = 0.17), and HD IL2 (*p* < 0.05) (Fig. 4). Increasing IL-2 doses to RUPP rats caused a dose-dependent decrease in placental PPET (Fig. 4a). Similar trends were observed in the renal cortex, where RUPP PPET-1 expression was elevated 8-fold compared to NP rats (Fig. 4b) and was significantly lowered with IL-2 treatment (*p* < 0.05).

### The effect of IL-2 infusion on circulating sFlt-1 in RUPP rats

**Fig. 5:**
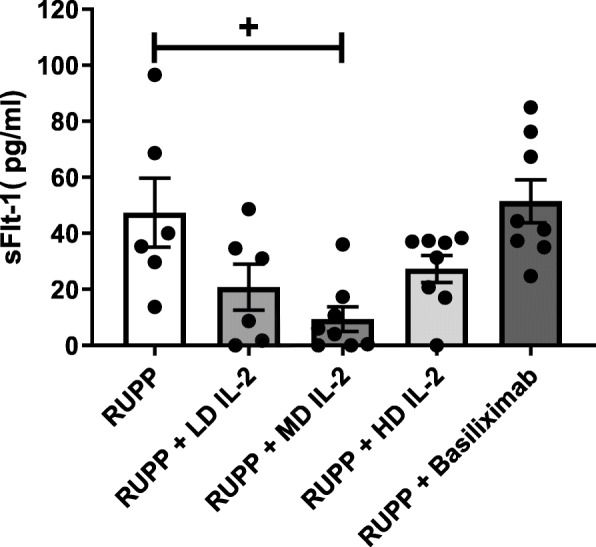
Circulating sFlt-1 in RUPP control rats and RUPP rats treated with increasing doses of IL-2. sFlt-1 was lowered with all three doses of IL-2, but only reached significance with the MD. Sflt-1 was 47.34 ± 12 pg/ml in RUPP rats (*n* = 6); RUPP + LD IL-2 sFlt-1 levels were 20.82 ± 8 pg/ml (*n* = 6), 9.34 ± 4.37 pg/ml (*p* < 0.05 vs RUPP) in RUPP + MD IL-2 (*n* = 8), and was 27.32 ± 5 pg/ml in RUPP + HD IL-2 (*n* = 8). There was no change with basiliximab treatment in RUPP rats (*n* = 8) compared to RUPP controls

We have previously published sFlt-1 to be elevated in RUPP rats compared to NP rats. Therefore, for this study, we focused on the effect of IL-2 treatment on sFlt-1 in RUPP rats. Sflt-1 was 47.34 ± 12 pg/ml in RUPP rats and was lowered with all three doses of IL-2, but only reached significance with the MD. RUPP + LD IL-2 sFlt-1 levels were 20.82 ± 8 pg/ml and 9.34 ± 4.37 pg/ml (*p* < 0.05 vs RUPP) in RUPP + MD IL-2 and were 27.32 ± 5 pg/ml in RUPP + HD IL-2, (Fig. 5).

### The effect of IL-2 inhibition on blood pressure and fetal weight in RUPP and NP

There was no difference in blood pressure between NP and NP + basiliximab (101 ± 2 vs 111 ± 5, ns, *p* > 0.99), or RUPP and RUPP + basiliximab (124 ± 2 vs 123 ± 3, ns, *p* > 0.99) (Fig. 1). There were no differences in pup or placental weights between NP and NP + basiliximab (*p* > 0.99) or between RUPP and RUPP + basiliximab (*p* > 0.99). Importantly, RUPP + basiliximab pup weights were not significantly less than NP + basiliximab (*p* > 0.99) which could indicate positive effects on fetal growth due to the reduction of IL-2-induced NK cells in the placenta. However, basiliximab significantly decreased the number of live pups in the RUPP + basiliximab group (*p* < 0.05) (Table 1). Consistent with previous studies, pup and placental weights were significantly lower in RUPP compared to NP (*p* < 0.05) (Fig. 2).

### The effect of IL-2 inhibition on natural killer cell activation in RUPP and NP

Activated circulating NK cells were significantly decreased in RUPP + basiliximab vs. RUPP rats (*p* < 0.05) (Fig. 3a). Total placental natural killer cells were significantly decreased in RUPP + basiliximab vs RUPP rats (*p* < 0.05) (Fig. 3b). This data suggests that even noncytolytic NK cells were decreased in the RUPP + basiliximab which may account for increased fetal reabsorption and decreased survivability (Table 1).

### The effect of IL-2 inhibition on preproendothelin expression in the placenta and kidney during pregnancy

Due to a lack of change in blood pressure in RUPP + basiliximab compared to RUPP control, PPET-1 was not measured in this arm of the study.

### The effect of IL-2 inhibition on circulating sFlt-1 in RUPP rats

RUPP sFlt-1 levels were 47.34 ± 12 pg/ml and were unchanged with basiliximab (RUPP + basiliximab 51.42 ± 7.71 pg/ml).

## Discussion

Clinical and preclinical studies have demonstrated a significant increase in cytolytic NK cells in PE, stimulating many questions as to the relevance of these cells in the pathology of this disease [6, 31–34]. We have previously shown the importance of NK cells in the pathophysiology of hypertension in the RUPP rat [1–3, 5, 6, 32]. NK cell proliferation and cytolytic activity can be stimulated by IL-2 at high concentrations which is an essential component of various metastatic cancer therapies [21]. However, at lower doses than those used for metastatic cancer therapy, IL-2 stimulates T reg cells to control NK cell proliferation and cytotoxicity. IL-2 is an important cytokine secreted by T cells that act not only on the T cells secreting it but also on surrounding inflammatory cells. Therefore, at low concentrations IL-2 communicates with T cells to decrease production of endogenous IL-2 and shut down the inflammatory response. We have previously shown that RUPP rats have elevated effector T cells, NK cells, and IL-2 compared to normal pregnant rats [21]. Therefore, this study was performed to examine the effects of IL-2 not only on NK cells, but also on blood pressure, pup weight, and vasoactive factors during pregnancy and in response to placental ischemia. The role of IL-2 during pregnancy was examined in two ways; first, we examined a role for IL-2 infusion to act through T regs and quell the inflammatory and vasoconstrictor response in RUPP rats. Importantly, IL-2 decreased blood pressure and cytolytic NK cells in the placenta of RUPP rats. Although IL-2 did not affect pup weights in normal pregnant rats, each dose of IL-2 resulted in even lower pup weight in RUPP rats with the MD and HD resulting in lower placental weight in RUPP rats. Although IL-2 decreased cytolytic NK cells, the lowered pup weight in RUPP rats indicates that either cytolytic NK cells are not the sole culprit for fetal demise in response to placental ischemia or that other factors were stimulated that caused further fetal demise in the IL-2 treated RUPP rats and this is being further investigated in our laboratory.

Preproendothelin is the precursor for endothelin (ET-1), a potent vasoconstrictor and major player in hypertension in response to placental ischemia. We have previously shown ET-1 to be stimulated in response to TNF-α and to play an important role in hypertension associated with elevated TNF-α during pregnancy [35]. In contrast, ET-1 was shown to be downregulated in response to IL-17 induced hypertension during pregnancy. This was further demonstrated in vitro during experiments in which we showed a dose-dependent effect of increasing concentrations of IL-17 to lower ET-1 secretion from HUVECS in culture [36]. IL-2 infusion into RUPP rats, like IL-17, significantly decreased PPET expression in both kidney and placenta compared to RUPP rats. Due to a lack of change in MAP in RUPP + basiliximab, PPET was not measured in this group. The exact mechanism of how IL-2 decreased PPET is unknown; however, IL-2 along with several other cytokines, such as IL-17 and TNF-α, and the autoantibody to the AT1 receptor (AT1-AA) are abundant in preeclampsia, and all have some effect to regulate ET-1, which except for IL-17, corresponds with their effect on blood pressure [37–40].

Placental ROS was measured by chemilumenescent assay in placentas from RUPP and RUPP + basiliximab, and no differences were found among these groups and therefore these data were not included. Several studies from our lab have shown that NK cell activation can lead to increased mitochondrial dysfunction and reactive oxygen species in both the kidney and placenta, which contribute to end organ damage and hypertension in pregnant rats [6]. Studies examining a role for IL-2 to preserve mt function by lowering NK cells in the kidney and placenta are under way in our laboratory. In addition, we have previously shown sFlt-1 to be increased in RUPP compared to NP rats. Although IL-2 reduced sFlt-1 in RUPP rats, the MD dose was the only one that significantly reduced sFlt-1 in RUPP rats (Fig. 5). Basiliximab had no effect on circulating sFlt-1

The original intentions of this study were to demonstrate a role for IL-2 blockade to lower MAP and cytolytic NK cells and improve pup weight in RUPP rats. Therefore, we utilized IL-2 inhibition with basiliximab in RUPP rats. To our surprise basiliximab administered to RUPP rats had no effect on blood pressure but did lower circulating and placental activated NK cells. Moreover pup weights were not significantly less than that of NP + basiliximab which suggest that there could have positive fetal effects. However, when we calculated fetal survivability, we found it to be even lower with basiliximab than with placental ischemia alone; however, this did not reach statistical significance. It could be that blocking IL-2 in placental ischemic rats suppresses total NK cell activation, including those that were noncytolytic that could have played some protective role for the fetal environment.

Overall this study suggests that IL-2 infusion into RUPP rats was protective for the mother, via a decrease in activating NK cells, the vasoconstrictor ET-1, sFlt-1, and maternal hypertension in response to placental ischemia. However, infusing IL-2 caused additional stress or harm to the growing fetus, as evidenced by reduced placental weights in NP rats and further reductions in fetal weight observed in placental ischemic rats treated with IL-2. Albeit this may not be a response to IL-2, but could have been a result of the lower blood pressure, or caused by another stimulus that was not identified in our study. Moreover, interfering with IL-2 signaling with basiliximab had positive outcomes on NK cell activation, and did not further reduce sFlt-1 or pup weight in RUPP rats, but did reduce the total number of pups in RUPPs treated with basiliximab indicating further harm to the fetal milieu. Moreover, basiliximab was not effective in lowering maternal blood pressures in the presence of placental ischemia

Although this study is novel and tested a dose response of IL-2 in both NP and RUPP rats, along with treatment of IL-2 inhibition via basiliximab, more studies are warranted. One of the major limitations of this study is the low numbers of rats in each group. A second limitation is that the determination of the inflammatory profile of the blood and placenta of these rats was limited to NK cells and T regs only. Our future studies in which we are determining the effect of IL-2 on mt stress will include examining levels of circulating proinflammatory cytokines.

In conclusion, IL-2 infusion demonstrated beneficial effects to decrease activated NK cells and significantly lower blood pressure in placental ischemic rats but also caused a reduction in fetal weight in the presence of placental ischemia. Moreover IL-2 significantly lowered ET-1, and MD IL-2 lowered sFlt-1, which indicates a mechanism whereby IL-2 had an antihypertensive effect. Importantly, interruption of IL-2 signaling with basiliximab had no positive outcomes for mother or the collective number of fetuses. Therefore, we conclude that IL-2 may be an important target with antihypertensive therapeutic potential for PE; however, a better understanding of its role in healthy pregnancy, especially fetal growth and development, should take precedence and should first be further examined. This study demonstrates the importance of IL-2 and nonactivated NK cells for fetal development and supports the notion that we still have much to learn about the role of inflammatory factors contributing to the complexity of healthy pregnancy outcomes.

### Perspectives and significance

IL-2 is a cytokine secreted by TH1 cells that proliferates, activates, and mobilizes T cells and natural killer cells. IL-2 is increased in RUPP rats; however, blockade of IL-2 in RUPP rats had no effect to improve maternal hypertension or fetal weights. IL-2 supplementation to normal pregnant rats had no effect on maternal hypertension or fetal weights. However, IL-2 administration reduced placental weight. Importantly, low dose IL-2 infusion lowered blood pressure in RUPP rats but also lowered fetal weight. Moreover, basiliximab may have worsened fetal outcomes in response to placental ischemia and should be investigated prior to consideration as a therapeutic for PE. In summary, IL-2 infusion demonstrated beneficial maternal effects to decrease activated NK cells and significantly lower blood pressure in placental ischemic rats, but may have negative outcomes for the offspring.

## Data Availability

Please contact author for data sets

## Abbreviations

IL: Interleukin
HD: High dose
MD: Middle dose
LD: Low dose
PE: Preeclampsia
CD: Cluster determinant
NP: Normal pregnant
RUPP: Reduced uterine perfusion pressure
